# Body Composition, Muscle Strength, and Physical Function of Patients with Bethlem Myopathy and Ullrich Congenital Muscular Dystrophy

**DOI:** 10.1155/2013/152684

**Published:** 2013-09-12

**Authors:** Maria Teresa Miscione, Francesca Bruno, Claudio Ripamonti, Giuliana Nervuti, Riccardo Orsini, Cesare Faldini, Massimo Pellegrini, Daniela Cocchi, Luciano Merlini

**Affiliations:** ^1^Department of Orthopaedics, Istituto Ortopedico Rizzoli, IRCCS, 40136 Bologna, Italy; ^2^Department of Statistical Sciences, University of Bologna, 40126 Bologna, Italy; ^3^Medicina Generale, Istituto Ortopedico Rizzoli, IRCCS, 40136 Bologna, Italy; ^4^Direzione Sanitaria, Istituto Ortopedico Rizzoli, IRCCS, 40136 Bologna, Italy; ^5^Department of Public Health Sciences, University of Modena and Reggio Emilia, 41122 Modena, Italy; ^6^Laboratory of Musculoskeletal Cell Biology, Istituto Ortopedico Rizzoli, IRCCS, 40136 Bologna, Italy

## Abstract

*Objective.* To determine the contributions of body mass, adiposity, and muscularity to physical function and muscle strength in adult patients with Bethlem myopathy (BM) and Ullrich congenital muscular dystrophy (UCMD). *Materials and Methods.* Evaluation involved one UCMD and 7 BM patients. Body composition was determined by body mass index (BMI) and dual-energy-X-ray-absorptiometry (DXA), muscle strength by dynamometry, physical function by the distance walked in 6 minutes (6MWD), forced vital capacity (FVC) by a spirometer. *Results.* Six participants were of normal weight and 2 overweight based on BMI; all were sarcopenic based on appendicular fat free mass index (AFFMI); and 7 were sarcopenic obese based on AFFMI and % fat mass. Average muscle strength was reduced below 50% of normal. The 6MWD was in BM patients 30% less than normal. FVC was reduced in 4 of the BM patients. Muscle strength had a good correlation with the physical function variables. Correlation between muscle strength and BMI was poor; it was very high with AFFMI. AFFMI was the best single explicator of muscle strength and physical function. *Conclusion.* Muscle mass determined by DXA explains most of the variability of the measures of muscle strength and physical function in patients with BM and UCMD.

## 1. Introduction

Mutations in any of the three genes (COL6A1, COL6A2, and COL6A3) coding for collagen VI, a major extracellular matrix protein of the endomysium of skeletal muscles, cause the collagen VI-related myopathies [1] including Bethlem myopathy (BM), Ullrich congenital muscular dystrophy (UCMD), and the two rarer variants limb girdle and myosclerosis myopathy [2, 3]. Bethlem myopathy is a congenital or early-onset muscular dystrophy characterized by axial and proximal muscle weakness [4, 5], and the hallmark of the disease is the presence of contractures of the interphalangeal joints of the last four fingers [6]. Bethlem myopathy is usually mild, sometimes slowly progressive, with some affected individuals over 50 years of age needing aids for outdoors mobility [7]. Respiratory failure is part of the clinical spectrum and can occur in patients with preserved ambulatory function [8]. UCMD is a severe congenital muscular dystrophy characterized by early onset, generalized and rapidly progressive muscle wasting and weakness, proximal joint contractures, and distal joint hyperflexibility. Independent ambulation is not achieved in the severe cases or is lost during childhood/adolescence in most cases [9–11]. Respiratory failure is early and progressive and may require artificial ventilatory support in the first or second decade of life [7]. 

In the EU, a rare disease is one which affects fewer than 5 people per 10,000 [12]. COL6 myopathies, although probably under diagnosed [13], are very rare disorders with the prevalence estimated as 0.77 per 100,000 for BM and 0.13 per 100,000 for UCMD [14].

The turning point in basic research on collagen VI myopathies was the discovery that mitochondrial dysfunction mediated by inappropriate opening of the permeability transition pore plays a key role [15–18]; defective autophagy with impaired removal of defective mitochondria amplifies the defect [19]; and reactivation of autophagy with a low-protein diet or treatment with cyclosporine A, the mitochondrial PTP inhibitor, cured Co6a1^−/−^ mice [15], hinting at a common target among all beneficial treatments—namely, autophagy [19, 20]. 

From a scientific and clinically relevant standpoint, the identification, measurement, and treatment monitoring of muscular dystrophy using a single easily administered cost effective test or measure is not currently possible [13]. Therefore, in clinical trials the methodological approach should be as comprehensive and multidimensional as possible, possibly evaluating the same aspect of skeletal muscle with different tools/instruments, to obtain confirmatory evaluations to findings [21]. Clinical trials have already involved paediatric patients with UCMD [17, 18] and planned for adult patients with collagen type VI myopathies aiming at correcting defective autophagy [22, 23]. Muscular dystrophy is characterized by an interconnected decline in muscle mass, muscle strength, and muscle function. Therefore, it would be mandatory to include a test of all these aspects as the best assessment in clinical trials. The purpose of this study is to determine the relative contributions of body mass, adiposity, and muscularity to measured physical function and muscle strength in adult patients with COL6 related myopathies.

## 2. Materials and Methods

We reviewed the records of our 75 patients with a clinical/laboratory phenotype compatible with the diagnosis of BM/UCMD [7]. Forty-four had a definite molecular diagnosis with recognition of a pathogenetic mutation/s in one of the three COL6 genes [3, 24–30]. Of the 27 patients, aged 18 years or more, 22 had a diagnosis of BM, 3 of myosclerosis myopathy, and 2 of UCMD. Fourteen of these, who have been followed in the previous 12 months, were contacted. 8 patients (5 female, 3 male, mean age 31 ± 9) with collagen type VI related myopathies accepted to be included in this study. The 7 BM patients were ambulatory, while the one with UCMD was never able to walk and was on nocturnal noninvasive ventilation. The study protocol was approved by the institutional ethical committee. All subjects were fully informed and gave their written informed consent.

Body composition was obtained by DXA (Hologic 4500 W; software version 11.2; Hologic, Inc., Waltham, MA). According to the tree-compartment model of body composition the Hologic software provided regional and whole body estimation of lean mass (LM), fat mass (FM), and bone mineral content (BMC). BMC and LM were added to obtain fat free mass (FFM). Appendicular fat free mass (AFFM) represents the sum of both arms and legs [31]. Body mass, FM, FFM, and AFFM were normalized to height 2 as control for skeletal size [32]. Women with appendicular fat free mass index (AFFMI) <5.45 and men <7.26 were classified as sarcopenic [31, 32]. BMI, an index of obesity, was derived from body mass measured by DXA to the nearest gram and height measured to the nearest 0.1 cm. We used BMI to categorized participants as obese (BMI ≥ 30), overweight (25 ≤ BMI < 30), or normal weight (BMI < 25) [33]. In addition subjects were classified as obese if their percentage body fat derived from DXA was above the 90th percentile of the Italian population [34]. For men, this cutpoint was 30% body fat; for women, it was 41% body fat [34]. Maximal isometric strength was assessed using a hand-held dynamometer (Type CT 3001, Citec, C.I.T. Technics BV, Groningen, The Netherlands) [35]. Four muscle groups were examined bilaterally: hand grip, elbow flexors, knee extensors, and knee flexors [36–38]. Each individual muscle group was tested for at least 3 seconds using a “make” test [37]. The maximum force from three attempts was used in analysis. A composite score (megascore) was calculated by summing the maximal force of the 8 tests for each patient [38, 39]. Forced vital capacity (FVC) was determined with an electronic spirometer, and per cent-predicted values were calculated based on normal published values. A value between 50% and 70% was considered moderately reduced; a value less than 50% was considered severely reduced [8]. The 6 minute walk test (6MWT) was performed in a corridor, and standardized verbal instructions were given every minute. Timed tests include the time to walk 10 m and to climb 4 steps.

## 3. Statistical Analysis

The very limited sample size suggested using simple linear regression models to determine the separate relative contributions of the various indices. Linear correlation coefficients have, therefore, been computed for various couples of variables with the aim of choosing the variables for simple linear models without intercept. High values of the linear correlation coefficients help in the choice of identifying the variables with potentially similar meaning for choosing both the dependent and the explanatory variables. The separate relative contributions of body mass and muscularity (seen as explanatory variables) to muscle strength, 6MWD, and pulmonary function (i.e., dependent variables) were evaluated via linear-regression models, expressed as *y*_*i*_ = *βx*_*i*_ + *e*_*i*_, without intercept. Each *β* coefficient was estimated via ordinary least squares. The values of this coefficient denote the increment/decrement of the dependent variable for a unit increment of each explanatory variable. To evaluate the strength of each simple linear relationship, we used the goodness of fit measure *R* ^2^. Measurable variables are presented as mean (*x*)  ± standard deviation (SD) and categorical data as number and percentage.

## 4. Results

### 4.1. Body Composition

6 participants were of normal weight and 2 were overweight based on BMI, all were sarcopenic based on AFFMI, and 7 were sarcopenic obese based on AFFMI and % fat mass (Table 1). FFMI was well below the 5th percentile for all the patients as compared to the normal age related population and also to the 70–80-year-olds [34]. The sex differences in body composition were as expected, with women having similar BMI but significantly greater adiposity and less muscularity than men.

### 4.2. Muscle Strength and Physical Function

Muscle strength (Table 2) was reduced in all muscle groups compared with the normative values [35, 40]. In particular, knee extension and elbow flexion were the weakest compared with healthy subjects in which knee extension exceeds in all 250 N, and elbow flexion exceed 150 N in women and 250 N in men [35, 40].

The mean 6MWD was in the 7 walkers 415 ± 71 m (418 ± 38 m in women and 411 ± 105 in men) as compared to 593 ± 57 and 638 ± 44, respectively, in women and men healthy Italian subjects in the same age range [41]. In BM patients %FVC was moderately (<70%) reduced in three and severely reduced (<50%) in one. The UCMD patient with a FVC of 14% was on mechanical ventilation.

### 4.3. Correlation between Body Composition, Muscle Strength and Physical Function

All the muscle strength measures had a good correlation with the physical function variables (Table 3). The megascore, in particular, showed very good linear correlation with all the physical function variables (0.72–0.92), stressing its value of muscular strength synthesis. The single measures of physical strength exhibited more variable values (0.38–0.96). Hand grip, in addition to being an important component of megascore and the most easy to perform and repeatable test, had a very strong linear correlation with FVC and %FVC (*r* = 0.96 and *r* = 0.89, resp.).

The linear correlation coefficients between couples of possible explicative variables of body mass and muscularity were different. Highest values, oscillating between 0.95 and 0.98, occurred between all measures (FFM, FFMI, AFFM, and AFFMI) derived by DXA but were very low between BMI and all these DXA measures of muscularity (0.08–0.17).

The indices of adiposity, BMI and FMI, had a negative correlation with all the muscle strength and physical function variables; BMI correlation coefficients, in particular, were very low and close to independence (Table 4). On the contrary, the indices of muscularity FFMI and AFFMI showed strong positive values when associated with all the dependent variables (Table 4). The highest values of linear correlation occurred between measures of muscle strength and particularly the megascore and FFMI (*r* = 0.87), and AFFMI (*r* = 0.93), indicating in this case a strong linear association and not a mere replication of the same information. 

Body compositions measures (Table 5), expressed alternatively via BMI and AFFMI, behaved very similarly when proposed as linear explicators of indicators of muscle strength (megascore and hand grip) and physical function (6MWD and %FVC). BMI was a good linear explicator, with *R* ^2^ values varying from 0.77 to 0.85; however, the best single explicator was AFFMI, with *R* ^2^ values varying from 0.90 to 0.93.

## 5. Discussion

The main objective of this study was to measure and correlate body composition, muscle strength, and physical function in a cohort of patients with collagen VI related myopathies. 

Body composition was evaluated with BMI and DXA. Body fat was greatly underestimated by BMI that classified only 2 patients as overweight while all but one resulted obese according to their percentage of body fat derived by DXA. In addition, DXA showed that all patients had a great reduction in muscularity both in the total body as indicated by the FFMI and particularly in the limbs with the AFFMI so that all could be classified sarcopenic [31, 32]. It should be stressed that BMI determinations do not measure body fat directly nor distinguish between fat and lean (nonfat) body mass. In fact, in patients there was no evident linear relationship (correlation coefficients ranging from 0.08 to 0.17) between BMI and the measures of muscularity derived by DXA. BMI correlates to direct measures of body fat, such as underwater weighing and DXA for most normal people [42, 43]. However, DXA, providing whole body and regional estimation of skeletal muscle mass *in vivo* [44], is particularly relevant for the evaluation of body composition in neuromuscular patients who are undermuscled to body size [45–48].

Muscle strength, measured with a dynamometer, was diffusely reduced with average values less than 50% of normal in the tests of knee extension, hand grip, and elbow flexion. 

All the measures of physical functions were reduced in these patients. The distance walked in 6 minutes was in the 7 BM patients 30% less than in normal age-matched subjects [41]. %FVC was reduced in 4 of the 7 BM, moderately (<70%) in 3, and severely in one (<50%). In a cohort of 43 BM patients, FVC was found moderately reduced in 7 (16%) and severely reduced in 2 [8]. Our figures further stress the importance to regularly evaluate the respiratory function not only in UCMD but also in BM patients [8, 10, 49]. 

Muscle strength was positively correlated with the measures of physical function. The megascore showed strong correlation with all the physical functions, respiratory functions (0.78–0.89), time to climb 4 steps (0.92), time to walk 10 m (0.72), and 6MWD (0.74). A good relationship between motor ability and muscle strength has been found in patients with neuromuscular disorders confirming that loss of function is due to loss of muscle strength [37, 50, 51]. Most myopathies, including collagen VI-related myopathies, are slowly progressive disorders characterized by muscle wasting and weakness that compromise motor and respiratory functions. If a cure is available, it should have a positive effect on muscle strength [13].

The various indices of body composition showed very different correlation with the measures of muscle strength and of physical function. It should be noted that muscle mass is the primary determinant of muscle strength in normal subjects [52, 53]. This statement was also true in our patients, in whom the indices of muscularity (FFMI and AFFMI) were strongly correlated with muscle strength. AFFMI showed the highest coefficients of correlation with muscle strength (0.76 with hand grip and 0.93 with megascore), 6MWD (0.52), timed tests (0.49 and 0.78), and pulmonary functions (0.68 and 0.86). Interestingly, the correlation between AFFMI and 6MWD was only moderate indicating that the contribution of muscularity explains only 50% of the performance in the 6MWD. In healthy subjects, it has been shown that age, height, sex, and weight were independent contributors to the 6MWD, thus, explaining up to 66% of the variability [54–56]. Finally, AFFMI was also the best single explicator of the measures of muscle strength and physical function. 

## 6. Conclusions

Collagen type VI related myopathies, like most muscular dystrophies, show a wide range of clinical severity that is usually evaluated with the assessment of muscle strength and physical function. The best assessment in clinical trials should be as comprehensive and multidimensional as possible in order to fully explore the interconnected compromise of muscle mass, muscle strength, and function. The evaluation of body composition, in particular of muscle mass with DXA, explaining most of the variability of the measures of muscle strength and physical function, should be included in clinical trials.

## Figures and Tables

**Table 1:** Body mass, adiposity, and muscularity. Summaries of individual data grouped according to sex.

Variable	Women (*n* = 5)	Men (*n* = 3)
Total mass (kg)	66.7 ± 12.1 (range: 45–74)	66.3 ± 11.1 (range: 55–77)
BMI (kg/m^2^)	22.9 ± 4.2 (range: 18–29)	22.3 ± 3.6 (range: 18–26)
% FM	51.6 ± 6.6 (range: 41–59)	34.6 ± 14.2 (range: 18–44)
FMI (kg/m^2^)	12.0 ± 3.6 (range: 7–17)	8.0 ± 4.0 (range: 3–11)
FFMI (kg/m^2^)	10.9 ± 0.8 (range: 10–12)	14.3 ± 1.3 (range: 13–15)
AFFMI (kg/m^2^)	4.2 ± 0.8 (range: 3–5)	5.8 ± 0.6 (range: 5–6)

BMI: body mass index, FM: fat mass, FMI: fat mass index, FFMI: fat free mass index, AFFMI: appendicular fat free mass index.

**Table 2:** Muscle strength and measured physical function. Summaries of individual data grouped according to sex.

Variable	Women (*n* = 5)	Men (*n* = 3)
Hand grip (N)	47.3 ± 18.8 (range: 12–73)	99.0 ± 22.8 (range: 67–119)
Elbow flexion (N)	38.8 ± 13.7 (range: 10–52)	99.3 ± 25.0 (range: 59–133)
Knee extension (N)	76.2 ± 38.2 (range: 24–136)	126.3 ± 77.6 (range: 50–225)
Knee flexion (N)	76.7 ± 31.5 (range: 18–107)	123.0 ± 55.4 (range: 54–185)
Megascore (N)	478 ± 199 (range: 160–680)	895 ± 286 (range: 651–1210)
10 m (1/*T*)	0.11 ± 0.07 (range: 0–0.17)	0.13 ± 0.04 (range: 0.09–0.18)
4 steps (1/*T*)	0.14 ± 0.09 (range: 0–0.22)	0.24 ± 0.09 (range: 0.17–0.33)
6MWD (m)	335 ± 190.2 (range: 0–458)	411 ± 104.5 (range: 320–525)
FVC (mL)	1788 ± 863 (range: 460–2670)	3687 ± 832 (range: 2760–4370)
% FVC	49 ± 23.0 (range: 14–72)	73.0 ± 17.3 (range: 53–84)

N: Newton, *T*: time, 6MWD: 6 minute walking distance, FVC: forced vital capacity.

**Table 3:** Linear correlation coefficients between muscle strength (lines) and physical function (columns) variables.

	6MWD (m)	10 m (1/*T*)	4 steps (1/*T*)	FVC (mL)	% FVC
Megascore (N)	0.74	0.72	0.92	0.89	0.78
Hand grip (N)	0.62	0.58	0.77	0.96	0.89
Elbow flexion (N)	0.45	0.38	0.62	0.94	0.81
Knee extension (N)	0.66	0.65	0.82	0.62	0.53
Knee flexion (N)	0.77	0.80	0.90	0.67	0.59

6MWD: 6 minute walking distance, *T*: time, FVC: forced vital capacity, N: Newton.

**Table 4:** Linear correlation coefficients between potential dependent (lines) and explanatory (columns) variables.

	BMI	FMI	FFMI	AFFMI
Megascore (N)	−0.06	−0.49	0.87	0.93
Hand grip (N)	−0.26	−0.59	0.71	0.76
Elbow flexion (N)	−0.10	−0.46	0.74	0.76
Knee extension (N)	0.07	−0.30	0.75	0.82
Knee flexion (N)	−0.03	−0.41	0.78	0.83
6MWD (m)	−0.13	−0.32	0.41	0.52
10 m (1/*T*)	−0.26	−0.44	0.40	0.49
4 steps (1/*T*)	−0.12	−0.45	0.68	0.78
FVC (mL)	−0.04	−0.42	0.78	0.86
% FVC	−0.02	−0.30	0.57	0.68

BMI: body mass index, FMI: fat mass index, FFMI: fat free mass index, AFFMI: appendicular fat free mass index, N: Newton, 6MWD: 6 minute walking distance, *T*: time, FVC: forced vital capacity.

**Table 5:** Linear models (*y*_*i*_ = *βx*_*i*_ + *e*_*i*_) of separate relative contribution of body mass and muscularity to muscle strength and physical function.

Dependent variables	Predictor variables	*β*	*R* ^2^
Megascore (N)	BMI	27.22	0.81
	AFFMI	141.05	0.94
Hand grip (N)	BMI	2.82	0.77
	AFFMI	14.64	0.91
6MWD (m)	BMI	15.52	0.82
	AFFMI	77.58	0.90
% FVC	BMI	2.50	0.85
	AFFMI	12.48	0.93

*R* ^2^: coefficient of determination, 6MWD: 6 minute walking distance, N: Newton, FVC: forced vital capacity.
